# Dromokaition Psychiatric Hospital of Athens: from its establishment in 1887 to the era of deinstitutionalization

**DOI:** 10.1186/s12991-015-0047-1

**Published:** 2015-02-15

**Authors:** Markella Fiste, Dimitrios Ploumpidis, Costas Tsiamis, Effie Poulakou-Rebelakou, Ioannis Liappas

**Affiliations:** Psychiatric Hospital of Athens Dromokaition, 84 Kallisthenous str., Athens, 11852 Greece; 1st Department of Psychiatry, Medical School of Athens, Athens, Greece; Department of Microbiology, Medical School of Athens, Athens, Greece; History of Medicine, Medical School of Athens, Athens, Greece; 2nd Department of Psychiatry, Medical School of Athens, Athens, Greece

**Keywords:** Dromokaition Psychiatric Hospital, Internment, Biological psychiatry, Psychosurgery, Occupational therapy, Deinstitutionalization, Private donation

## Abstract

Dromokaition Psychiatric Hospital opened its doors in 1887, following the donation made by Zorzis Dromokaitis from the island of Chios. Private donations and all forms of charities had contributed to a large extent in the establishment of hospitals across Greece, during the late 19th and the early 20th century. Dromokaition was one of them but it was also unique, as it was the first psychiatric hospital in Athens, admitting patients from every part of the country. This paper aimed at highlighting the long service of the institution through the different historical periods the country went through. We present the chronicle of its foundation, the development of its inner structure, and the medical and organizational influences which it received, along the way. The therapeutic methods used during the first decades of its operation reflected the corresponding European standards of the time. As a model institution from its foundation, it followed closely the prevailing European guidelines, throughout its historical path, either as an independent institution or as an integrated one within the National Health Service.

## Introduction

In the modern history of Greece, the first hospital-asylum for mental illnesses was founded in 1838 on the island of Corfu, at the time under the rule of the British High Commission. This was a milestone for the whole region, influenced by the asylum model, prevailing in Europe at the time; it was the asylum era for European psychiatry after all. In 1864, Corfu and the rest of the Ionian Islands were incorporated to the fledgling Greek State, following the Unification Treaty. Thus, the Corfu Mental Institution became the first of its kind in the country, serving mainly the local population, as depicted in the majority of its inpatients. Nevertheless, a low percentage of the latter came from other parts of Greece, including its capital, Athens.

A not unusual practice in Greece, at the time, was to care for mentally ill patients in monasteries and churches, in low numbers though. The only Greek general hospital admitting mentally ill patients was located in the Island of Chios. To this should be added the Greek hospitals of Smyrna and Constantinople, which were then part of the Ottoman Empire.

Dromokaition Hospital was founded at a most convenient time. It was a time when the need for the care of the mentally ill in the capital was a subject of attention and concern by the Athenian society [1,2]. A rich benefactor from the island of Chios, Zorzis Dromokaitis (1805–1880), provided the necessary funds for the purpose. At the same time, he also appointed a committee to ensure the execution of his will in the most appropriate way. The changing needs of the times and the demanding management of the donations and charities compelled the committee to appoint an administrative board and set forth regulatory rules to ensure a smooth, adaptable, and flexible operation of the institution.

Throughout its historical path, the institution was profoundly affected by all major events that took place concurrently in the history of the country: In the 1920s, the considerable increase of the destitute patients, following the Greco-Turkish War and the Asia Minor Catastrophe; in the 1940s, there was the considerable decrease in all subsidies and donations, the aftermath of the Second World War.

It should be noted though that, while a number of state institutions agreed and signed loan contracts from the state, the institution decided not to follow suit. In a breakthrough move, it opted for an independent course, relying on donations and on the bequest’s funds.

Dromokaition Hospital was an independent institution from its inception, a policy which continued even after its incorporation to the National Health Service in 1983 and the deinstitutionalization programs from 1984 to 2009 [3]. Nevertheless, from the mid-2000s and onwards, it faces a relentless policy from the State, aiming at the encroachment of its independence and in total disagreement to its founder’s will.

### The establishment of Dromokaition and its organizational plan

The hospital opened its doors on 1st October 1887 in the region of Dafni, with a total of 26 patients admitted by the end of the year [4]. As mentioned above, Dromokaition Hospital was a private institution, bequeathed by the rich merchant Zorzis Dromokaitis, from the island of Chios, to whom it also owes its name. The funding of the institution relied solely on charities, as dictated by its founder’s will. Its legal status as a privately run organization was approved and supervised directly by the Ministry of Internal Affairs, thus operating under its auspices.

The hospital was admitting private patients, who could afford their stay expenses, offering three levels of accommodation facilities, i.e., first, second, and third class, in decreasing order of facilities. A low number of destitute patients were also admitted in the third class wards [5]. Thus, patients from different social strata and origin were served, including Greek expatriates. The major places of origin of patients were Central Greece, Peloponnesus, and Aegean Sea Islands, as well as five patients from abroad.

The number of patients admitted steadily increased, reaching 121 by the end of 1900. According to the French psychiatrist Lucien Libert, in the early 1900s, 100 new patients were admitted and an overall of 300 would be treated in Dromokaition every year [6,7]. An increase is also evident, especially from 1915 and thenceforth, in the number of patients admitted (Table 1). This is the effect of the Greco-Turkish War, the Asia Minor Catastrophe, and the two World Wars.

**Table 1:** **Admissions by sex and outpatient statistics from 1887 to 1949**

	**Admissions by sex**		**Total number**	**Cure**	**Improvement**	**No change**	**Death**	**Total**
	**M**	**F**						
1887–90	170	42	212	40	22	38	32	132
1891–95	249	87	336	51	84	99	61	295
1896–00	354	117	471	86	53	181	95	415
1901–05	471	170	641	123	51	312	111	597
1906–10	308	119	427	84	45	146	91	366
1911–15	248	95	343	60	47	104	102	313
1916–20	444	171	615	122	60	213	177	572
1921–25	611	263	874	209	19	334	199	761
1926–30	587	336	923	172	50	366	210	798
1931–35	605	315	920	234	0	460	187	881
1936–40	560	296	856	255	0	316	228	799
1941–45	580	389	969	277	0	382	599	1.258
1946–49	694	627	1.321	499	0	341	113	953

The executors of the benefactors’ will took all necessary measures to ensure that the institution operated in accordance and on par with the prevailing international standards. To this end, they adopted and implemented the therapeutic model endorsed by the French psychiatrist V. Lunier. This model included specific guidelines not only regarding the rules and regulations of the institution but also for the general architecture and the interior design of its buildings. The outcome was an institution that would function as a French Psychiatric Hospital of the late 19th century, i.e., a contemporary one for the time. The patients admitted were allocated to the different departments according to the nature of their mental illness and their concurrent physical ailments [8,9].

The great number of donations, made mostly by people from the island of Chios, made possible the placement of the patients to the appropriate wards. Particular attention was paid to the professional training and the general demeanor of the staff of the institution, in order to ensure the utmost level of care offered to the patients. This was achieved through compulsory training of all new staff and the organization plan implemented. The medical staff in particular was encouraged to attend important European medical meetings, conferences, and training seminars, when available, to keep an up-to-date level of knowledge and clinical practice.

A thorough clinical assessment and the establishment of a soothing doctor-patient relationship were both highly regarded issues by the institution’s directors. To this effect, the latter made every effort to ensure that they were both not only strongly implemented but also deeply entrenched into its mode of operation. This remained so throughout the institution’s historical path[10].

All necessary measures were taken in the acute phase of the admission, to ensure a humane but also effective management of the disturbed state of the patient. These were achieved via the implementation of standard operating procedures, which included a special isolation room and the use of other restraining measures like the infamous straightjacket and compulsory bed confinement. All the procedures employed were in accordance with the prevailing European practices [11,12].

All the modern, at the time, therapeutic approaches were employed, namely music therapy, walk with the assistance of a nurse, but also sports and entertainment activities. Occupational therapy was at the center stage of the therapeutic environment, aiming to maintain and improve, if possible, the patients’ employment abilities or even enhance them [13]. Involvement in the kitchen routine and food preparation procedures was one of the earliest practices, as was stock breeding [14]. The latter, apart from the beneficial effect on the patient, was a means of contributing to the food supply of the institution.

Artistic expression of the patients was strongly encouraged, helping to enhance the social integration of the patients and also ameliorate the consequences of the institutionalization, which was an inevitable evil of the long hospitalization. The patients, according to their wishes and inclinations, could attend workshops for painting, woodcraft, or pottery, always under the careful supervision of trained staff [15]. Time passed but the aforementioned methods never ceased to be employed. In particular, the music and painting workshops are still active, while the cooking and pastry ones are included in the daily routine activities of the therapeutic milieu in an inpatient but also in a number of outpatient facilities.

The Hydrotherapy Department of the institution offered a variety of therapeutic techniques. The hydrotherapies available included full or half body healing or plain (i.e., with and without a diluted substance), warm or cold. According to Michalis Gianniris (1865–1956), the second superintendent in the history of the institution, warm baths would have a soothing effect ‘on a sensitive nerve system and an over-aroused brain’. The patient would remain immersed for a period of 30 min to 3 h in each session, in warm water of constant temperature, depending on the doctor’s instructions [16]. The healing baths made use of a variety of solutes: sulfur grain, mercury, mustard, or saline diluted in water. During the cold baths, the patient was immersed in water, at 16°C to 22°C, for about 3 to 5 min, moving for the entire period. Another hydrotherapy method employed was the wrapping of the patient with a wet sheet, the temperature of which was specified by the doctor. The procedure was completed by two woolen blankets, which were put on top of the wet sheet [17]. Another treatment method in the Hydrotherapy Department was the shower baths.

Other forms of treatment also included blood-letting and a variety of special diets [18].

The combination of the antisyphilitic treatment in conjunction with the patients’ rest in a special sanatorium, a proper diet, and the avoidance of irritation was regarded as the best treatment option that could be offered at the time. Apparently, this was reflecting the current state of knowledge about the etiology, natural history, and clinical manifestations of the disease [19].

As early as 1895, Christos Tsirigotis (1841–1919), the first superintendant in the history of the institution, advocated in favor of official leave permits to hospitalized patients, stressing their beneficial, dual role of not only offering the psychiatrist valuable information but also motivating the patient to adopt desired behaviors (an early form of token economy). Although he made every effort to promulgate them, he was not successful, as they were not included in the mental health act of 1864, regulating the establishment of mental hospitals [20]. At the same time, he implemented the work for the patients, in the gardens of the institution, attributing to this beneficial properties, regarding the practice as a means of achieving ‘psychic decompression and mental elevation,’ in his words [21].

### Influences on the institution’s clinical and therapeutic practices

As discussed in the previous section, all the organizational and clinical practices adopted by the institution at any time, from its establishment and onwards, were in accordance with the prevailing trends in its European counterparts. Valentin Magnan’s doctrine of ‘degeneration’, permeating European psychiatry widely at the time, did not spare the institution, which during its first years adopted his views. Despite the controversies on his theories, Magnan undeniably represented the high water mark of the first biological psychiatry in France at the time, a fact the institution’s leadership could not ignore. Inevitably, the French nosology and classification were used. Over time though, the diagnosis of degeneration steadily dwindled, as the rubric of ‘dementia praecox’ took over in increasing numbers, reflecting the views of Emil Kraepelin. All the different diagnostic entities used by 1930 can be seen in Table 2.

**Table 2:** **Diagnostic categories used in 1930**

**Num.**	**Mental diseases**
1	Manic syndrome
2	Melancholic syndrome
3	Manic depressive psychosis
4	Paranoid and paraphrenic psychoses
5	Mental confusion
6	Psychosis due to degeneration
7	Schizophrenia and primary dementia
8	Progressive paralysis of the insane
9	Secondary dementia
10	Senile dementia
11	Presenile dementia
12	Organic disease of the brain
13	Epileptic psychosis
14	Hysteric psychosis
15	Alcoholic psychosis
16	Heroin mania
17	Psychosis due to cannabis

Schizophrenia was always a syndrome of utmost importance in psychiatry, of course under a variety of names, according to the historical period. The breakthrough in its description as nosological entity came from the work of Emil Kraepelin at the end of the 19th century. Although his ‘dementia praecox’ was a pivotal moment in the history of the disorder, it was Eugene Bleuler who coined its modern name in 1911.

In Dromokaition schizophrenia, with its relatively brand new name, made its nosological debut in 1926, dominating the diagnostic categories ever since, gradually replacing relative nosological entities like dementia praecox or insanity due to degeneration. The entity of ‘heroine-mania’ also made its appearance in 1930. A review of the nursing report logs and the statistics of the institution could help to delineate the emergence, time course, and demise of all different diagnostic trends in clinical practice at any time period.

#### Biological therapies

The discovery, in 1917, of the fever cure for neuropsyphilis using the malaria parasite was a breakthrough to the therapeutic nihilism dominating psychiatry for ages. For his discovery, Julius von Wagner-Jauregg (1857–1940) received the 1927 Nobel Prize in Medicine.

By 1925, the technique was practiced in the institution, not only for neuropsyphilis but also for several forms of psychosis. Apart from being cumbersome, the technique was expensive as well. The patients had to be infected with the right kind of malaria: tertian malaria, producing chills every third day. The situation improved as plasmodia were replaced with an oleaginous solution of sulfur or a typhus vaccine, thus making matters easier and less expensive.

In the 1930s, there was another import from Austria. It was the insulin coma therapy, introduced by Manfred Sakel in 1933 and adopted by the institution in 1936. The first to apply the treatment was Dr. N. Arcalidis (1901–1972), who had been trained for this abroad. A full physical examination was performed, before the application of the treatment, as well as a full gamut of investigations including liver function tests, a heart orthodiagram, electrocardiogram, and chest x-rays. At the first session, 20 units of insulin were administered, a dose that would increase steadily by 10 units per day, for the remaining sessions. Coma was induced with doses ranging from 60 to 200 units, and its duration would last for one and a half hours approximately. The number of the sessions would be determined by the clinical state of the patient. The overall period of treatment would not exceed 3 months in length, with a maximum number of patients treated no more than 12. A prerequisite for the process was the signing by the patients’ relatives of a form of informed consent [22]. A condition that allowed the institution to carry on with the particular form of treatment was that being a private hospital with the necessary budget; it could afford purchasing insulin directly from its production company, Welcome, in the UK, at a lower cost [23].

Soon after the introduction of insulin coma therapy, a second so-called shock therapy emerged. This was the cardiazol convulsive therapy introduced by a 38-year-old psychiatrist from Budapest, Ladislas von Meduna. In 1934, he proposed the amelioration of the symptoms of schizophrenia by putting the patients in a convulsive state. Thus, he introduced the convulsive treatment in 1935, using cardiazol, a compound that had been synthesized 10 years earlier and suggested to him by the Budapest professor of pharmacology. This was the formal begging of the era of the convulsive treatments. It differed crucially from the insulin coma therapy, where convulsions were not desired effect, and at the same time, it did not involve the induction of coma. As was the case with the insulin coma therapy, very soon the cardiazol treatment became available in the Dromokaition. It was introduced in 1936 by I. Skoulikidis (1884–1967) and N. Arkalidis, superintendent and assistant superintendent, respectively. Arkalidis had received training abroad, first in Vienna and later in Budapest, where Meduna was the Superintendent of the State Psychiatric Hospital. The technique consisted of injecting intravenously a bolus of 5 to 7 ml of cardiazol over a few seconds. The immediate effect of this was a sensation of fear and impending death, lasting up to 2 min, followed by loss of consciousness and the induction of seizure [24,25]. The signing of an informed consent form by the patients’ relatives was mandatory, in order to proceed.

The fame of both treatment methods soon faded. Although this was partly due to the serious concerns about their safety, the main reason though was the historic innovation by Ugo Cerletti (1877–1963) and Lucio Bini, in 1938: the electroconvulsive therapy. Its introduction to Greece was delayed due to the World War II, making its first appearance in 1945 [26]. In Dromokaition, it was first used by Dr. George Lyketsos (1916–2011), who received extensive training abroad in order to acquire all the skills and knowledge necessary, in order to apply the technique in the most efficient way. Lyketsos developed a research interest in the method, training doctors in it, writing articles, and publishing the results of his work. In 1946, he published his first work on the method, involving 100 cases [27]. An interesting finding was that of the 75 patients receiving six to 12 sessions, 12 of them exhibited what he recorded as ‘total recovery’ on their symptoms of catatonic schizophrenia. He regarded the method as superior to the cardiazol one, causing much less discomfort to the patient, as it was not associated with the feeling of fear and impending death synonymous with the latter. He also stressed its relative safety over it, as it displayed fewer complications: the milder convulsions resulted in fewer fractures, and there was also much fewer cases of thromboses and psychomotor excitation. Generally, he became an enthousiastic exponent of the method, which he deemed that it outclassed its cardiazol counterpart in every aspect, being safer, more effective, cheaper, and much simpler and quicker to apply [28,29]. As he recounts, the first electroconvulsive therapy appliance was built by him with input from his engineer friend A. Athanasoulas. He delivered the first session, with the assistant superintendent Arkalidis and a number of students filling the ECT suite. Despite his concerns, all went smoothly. The patient could recall his name and place of origin, so his relatives were found and he was eventually discharged from the hospital, where he had been confined for a number of years [30]. Lyketsos improved his skills and knowledge on the technique and used it extensively. It is reported that at a point, he achieved a personal record of performing 40 ECT sessions in 1 h [31]!

#### Psychosurgery

In the context of the success of the somatic treatments discussed above, it began to seem reasonable to the European psychiatric community to attempt to operate on the brain, so psychosurgical notions of the late 19th century reemerged. The modern history of psychosurgery begins in 1888, by the first operation carried out by a Swiss psychiatrist in Basel, Gottlieb Burckhardt. But it was the Lisbon neurologist Egas Moniz, who made the technique famous, earning a Nobel Prize in 1949 for this. It needs to be mentioned though that the first operations were carried out between 1935 and 1936 by the neurosurgeon Almeida Lima, operating under Moniz’s guidance. A further and crucial boost to the method was its adoption in 1946 and extensive use by the Americans Walter Freeman and David Watts, a neurologist and a neurosurgeon, respectively.

The method of prefrontal lobotomy was introduced in Dromokaition by G. Lyketsos and P. Kokkoris (1912–2006) in 1948. It was gradually abandoned though in the 1950s, following the international developments on the issue but also because of the advent of the first antipsychotics [32].

#### Progressive paralysis of the insane

It has been one of the main diagnoses of patients admitted in Dromokaition, especially in the first decades of 20th century. Numerous protocols of treatment have just underlined the incurability of the disease.

The pharmacological armamentarium of the institution included a variety a pharmaceuticals, in accordance with the international practices of the era. Phenobarbital and scopolamine were used as tranquilizers [33]. Chloral hydrate, trional, sulphonal, and opium were used as sedatives. Arsenic was used as a stimulant.

The decisive end to neuropsyphilis was brought about by the use of penicillin. Although it all began in 1927 at Oxford University by Alexander Fleming’s discovery of penicillin, the final and decisive part was written in ‘peripheral’ America, where penicillin was produced in large quantities, a problem that was creating a bottleneck to the wide use of the compound. Breaking this bottleneck was indeed a ‘peripheral’ American achievement first attempted at Peoria Illinois and eventually carried out at 21 different drug companies across US. Linking penicillin to syphilis was again an entirely American accomplishment, as John Mahoney, head of the Venereal Disease Research Center, started testing it in 1943 for the primary form of the disease. By 1944, it was being used for all the forms, including neuropsyphilis [34]. In Greece, the treatment was introduced in 1946 by Dr. Constantinos Choremis (1898–1966), who had traveled to the US for this purpose. The treatment was used in Dromokaition extensively, gradually overtaken by the advent of chlorpromazine.

### Opening of the doors of a psychiatric institution

A top priority for the psychiatrists of the institution was the introduction of all the therapeutic breakthroughs, which were transforming the psychiatric services in the United Kingdom, France, and Switzerland (Table 3). This effort intensified in the years after the Second World War [35].

**Table 3:** **Turning-points in the development of the mental therapeutic practices in Dromokaition**

**Date**	**Dromokaition**
19th–20th century	Corresponding European theoretical and practical approaches in mental illness treatment
1887	Emphasis on the inner organization of the mental hospital, its architecture, the staff’s behavior, the division of patients according to their mental condition and their placement in appropriate departments
1887–1900	Music therapy, a walk in the country under supervision, pool games, sports, work therapy, artistic expression, baths, enemas
1900	Hypnotic drugs, sedatives, stimulants, exsanguinations, special diets, anti-syphilitic treatment
1925	Fever therapy implemented in 1925
1936	Insulin shock therapy implemented in 1936
1936	Cardiazol convulsive therapy implemented in 1936
1945	Electroshock
1947	Dromokaition is the first mental hospital in Greece to open its gates, organize theatrical performances, film viewings, dance nights, recitals, concerts, festivities
1947	Work Therapy Department
1948	Prefrontal leukotomy implemented in 1948
1948	Choremis brought penicillin from America to Greece

In accordance with this stream of innovative efforts, Dromokaition became in 1947 the first Greek psychiatric hospital to allow its patients to walk freely within its grounds and later in the outside world [36,37]. A special department was created, which became the venue of many therapeutic activities. It offered reading and painting rooms [38], a technical game room, and to the delight of many, a cinema and a theater. Thus, a variety of activities could take place. These included concerts, recitals, choir singing, dancing, and other performances and festivities. The patients were allowed and encouraged to participate, after being selected according to their abilities, interests, and skills. It was at Dromokaition’s ancient style open theater where a cast by inpatients only performed a Greek tragedy [39]. During all these activities, there was a careful and detailed recording of the patients’ behavior, so the psychiatrists could have a detailed description of their patients’ functioning outside the ward, i.e., in a more ‘real world’ setting [40].

Despite the fact that the patient’s occupation with a variety of activities was an established and highly valued practice, the Occupational Therapy Department was formally established in 1947. Several types of activities and workshops were available. These included carpentry and textile craft, training in electrical and mechanical repairs, plumbing, constructions, shoemaking, printing and bookbinding, wickerwork, cutting and sewing, embroidery, knitting, cooking, painting, music, gardening, and even songbird care.

The advent of modern psychopharmacology in the 1950s transformed psychiatry forever, allowing the discharge of many patients, whose symptoms responded to the new treatments. The result was a significant reduction of the number of chronic patients [41]. The treatment outcome statistics, covering a period from 1887 to 1949 (Table 1), show that there was an annual increase on what was deemed a ‘cure’, first appearing after the first decade. The same applies to what was regarded as ‘improvement’. The disproportionate increase of the admissions, though, resulted in an increase in the percentage of the chronic patients.

The period from 1935 to the 1950s was marred by the war, what preceded it and its aftermath. The concomitant poverty and suffering affected the benefactors of the Institution; the result was a cutback in donations. The benefactors made up for it though in the following years, with intensive efforts and energetic work. In 1983, Dromokaition was integrated to the newly formed National Health Service. Although the hospital was operating with the rules and under the control of the Ministry of Health, the terms of the bequest safeguarded its independence, especially on administrative, managerial, and organizational matters [42].

1984 was a milestone year for the Greek psychiatric services, as funding from the European Union became available, in order to assist the deinstitutionalization efforts (Regulation 815/1984). With the help of the aforementioned funds, the institution expanded, real estate was bought, and new buildings were built in order to accommodate the new services. The latter included a day hospital, a psychotherapy center, a community mental health center, a vocational training department, and a residential house [43].

Deinstitutionalization was expedited through the implementation of the government’s ‘*Psychargos*’ plan, during the first and second phase of which, rehabilitation facilities were established. In Dromokaition, this was translated into the opening of boarding houses, residential homes, community flats, and vocational training centers.

Currently the institution’s role in the NHS has been upgraded. Its five acute admission wards, together with a psychogeriatrics one and a very important network of community facilities (which include two mental health centers, six residential houses, and a number of sheltered flats), make the institution a *sine qua non* of the state psychiatric services. The hospital is also on a 24-h on call service twice a week, covering Athens and a great part of the country, making it a pillar of the National Health Service.

Throughout its 127 years of existence, Dromokaition has faced many challenges, regarding especially its autonomy; many of them still go on today. As times change and with the government’s decision to close the remaining psychiatric hospitals, the need for the institution to transform and adapt to the new environment is both mandatory and vital. Any development though needs to take into account and respect accordingly, not only the integral terms of the benefactor’s will but also the views of the society in general and those of the users of the hospital’s services in particular.

## Conclusions

All the major events of the modern history of Greece had an impact on the institution, each leaving its mark. During its first years, it provided the standard institutional-asylum level of service for the mentally ill. Gradually, it succeeded in incorporating all new international developments and improvements in clinical practice and rehabilitation. A new era for the institution began in the 1980s, with its incorporation to the newly formed National Health Service.

The medical staff of the institution has made a considerable contribution to the medical research and literature of the country. An early example of this was the article by M. Gianniris and P. Sotiriadis in 1910, describing their observations on the Wasserman antibody test for the diagnosis of syphilis [20], following the discovery of *Treponema pallidum* by Fritz Schaudinn and Erich Hoffmann in 1905 [44].

From its early days, Dromokaition was acknowledged for the high standards of care it provided, thus making its services indispensible and well respected. This was corroborated by its outcome statistics over the years (Table 1), rendering it a *sine qua non* provider of psychiatric services in the country’s modern history, a fact evident during its private years period, but more so after its incorporation in the National Health Service, where it played a major role in the deinstitutionalization efforts [23] and still does so.

As times changed, it adapted accordingly to the demands of a changing nation. Nowadays, although the core of its administrative board consists of Greeks from the island of Chios, the selection of staff abides by the law for equal opportunities, so any Greek citizen can be employed.

At present, the institution has entered a new era, where reform and change are the prerequisites for its future. A golden mean needs to be reached though, where the implementation of all necessary reforms will not encroach upon the terms of its founder’s will.

## References

[1] Ploumpidis DN (1989). History of psychiatry in Greece, institutions, establishments and social framework. Thessaloniki: Synchrona Themata/Triapsis Logos. 1850–1920.

[2] Poulakou-Rebelakou E, Tsiamis C, Panteleakos G, Ploumpidis D (2009). The mentally ill as a “spectacle” in the Athenian streets. Historical and literary testimonies from the capital in the late 19th and the early 20th century. Archives of Hellenic Medicine.

[3] Dromokaition Sanatorium 1887-1987+15 (2001). A hundred and fifteen years of social service.

[4] Statistics of Dromokaition Mental Hospital in Dafni of the year 1888: Annual Report. Athens: National Printing and Lithography; 1889

[5] Lekka V (2012). History and theory of psychiatry.

[6] Kritsotaki D. Mental disease and psychiatric hospitalization. Social aspects and functions of psychiatry and psychiatric institutions in Greece and Scotland of the early 20th century. PhD thesis. University of Crete, History and Archaeology Department, Crete Island; 2009: 84.

[7] Ploumpidis DN (1989). History of psychiatry in Greece, institutions, establishments and social framework. Thessaloniki: Synchrona Themata/Triapsis Logos. 1850–1920.

[8] Gianniris M (1903). About hospitalization and treatment of the mentally Ill in mental hospitals or at home. On nurse training.

[9] Ploumpidis DN (1989). History of psychiatry in Greece, institutions, establishments and social framework. Thessaloniki: Synchrona Themata/Triapsis Logos. 1850–1920.

[10] Accountability and Statistics of the year 1894 of Zorzis and Tarsi Dromokaitou Mental Hospital under the protection of His Majesty the King: Annual Report. Athens: Private Press Perri brothers; 1895: 21–22

[11] Gianniris M (1903). About hospitalization and treatment of the mentally Ill in mental hospitals or at home. On nurse training.

[12] Karamanolakis V. The establishment of psychiatric institutions. Dromokaition Mental Hospital (1880–1903). M.A. thesis. University of Athens, History and Archaeology Department, Athens; 1997: 49.

[13] Regulation of function Dromokaition Sanatorium. Athens; 3/3/1975: 65.

[14] Fafaliou MS (1995). Iera Odos 343. Testimonies from Dromokaition.

[15] Regulation of function Dromokaition Sanatorium. Athens; 3/3/1975: 69.

[16] Gianniris M (1903). About hospitalization and treatment of the mentally Ill in mental hospitals or at home. On nurse training.

[17] Gianniris M (1903). About hospitalization and treatment of the mentally Ill in mental hospitals or at home. On nurse training.

[18] Gianniris M (1900). About progressive paralysis. Clinical and forensic study.

[19] Gianniris M (1900). About progressive paralysis. Clinical and forensic study.

[20] Fafaliou MS (1995). Iera Odos 343. Testimonies from Dromokaition.

[21] Karamanolakis V. The establishment of psychiatric institutions. Dromokaition Mental Hospital (1880–1903). M.A. thesis. University of Athens, History and Archaeology Department, Athens; 1997: 48.

[22] Statement of Consent from patient’s relative to implement treatment (Dromokaition Psychiatric Hospital of Athens Museum Archives).

[23] Fafaliou MS (1995). Iera Odos 343. Testimonies from Dromokaition.

[24] Lyketsos GK (1998). The novel of my life.

[25] Fafaliou MS (1995). Iera Odos 343. Testimonies from Dromokaition.

[26] Karavatos A, Ploumpidis DN, Christodoulou GN (2006). Anthology of Greek psychiatric texts.

[27] Lyketsos GK. Electroconvulsive therapy in Dromokaition Psychiatric Hospital. The first results over 100 cases. Kliniki 1946; 7.

[28] Lyketsos GK (1998). The novel of my life.

[29] Karavatos A, Ploumpidis DN, Christodoulou GN (2006). Anthology of Greek psychiatric texts.

[30] Lyketsos GK (1998). The novel of my life.

[31] Fafaliou MS (1995). Iera Odos 343. Testimonies from Dromokaition.

[32] Fafaliou MS (1995). Iera Odos 343. Testimonies from Dromokaition.

[33] Gianniris M (1900). About progressive paralysis. Clinical and forensic study.

[34] Shorter E (2009). History of psychiatry.

[35] Tzavaras A, Ploumpidis D, Asser A. Greek psychiatric patients during World War II and the Greek Civil War. International Journal of Mental Health Winter 2007–8. 1940–1949;36(4):57–66

[36] Dromokaition Sanatorium 1887-1987+15 (2001). A hundred and fifteen years of social service.

[37] Patelarou AE. Psychiatric facilities in 19th century Greece. M.A. thesis. University of Crete, Department of Medicine, Crete Island; 2012: 49.

[38] Kalofolias T (1954). Come along for a short tour “IN DROMOKAITION WITH THE NUTS”. Athenaiki..

[39] Dromokaition Sanatorium 1887-1987+15 (2001). A hundred and fifteen years of social service.

[40] Regulation of function Dromokaition Sanatorium. Athens; 3/3/1975: 70-1.

[41] Activity Report of Dromokaition Sanatorium 1957: Annual Report. Athens; 1958: 49

[42] Dromokaition Sanatorium 1887–1987 (2001). A hundred years of social service.

[43] Dromokaition Sanatorium 1887-1987+15 (2001). A hundred and fifteen years of social service.

[44] Dromokaition Sanatorium 1887-1987+15 (2001). A hundred and fifteen years of social service.

